# A Modified Lattice Configuration Design for Compact Wideband Bulk Acoustic Wave Filter Applications

**DOI:** 10.3390/mi7080133

**Published:** 2016-08-05

**Authors:** Qingrui Yang, Wei Pang, Daihua Zhang, Hao Zhang

**Affiliations:** State Key Laboratory of Precision Measuring Technology and Instruments, Tianjin University, Tianjin 300072, China; yangqingrui@tju.edu.cn (Q.Y.); dhzhang@tju.edu.cn (D.Z.)

**Keywords:** bulk acoustic wave (BAW) filters, wideband filters, modified lattice configuration, schematic design, electromagnetic (EM) simulation

## Abstract

High-performance bulk acoustic wave (BAW) filters have been widely applied in the advanced radio frequency (RF) wireless communication systems in the past decade. However, the demand for filters with large bandwidth, up to 10%, still puts a significant stress on the typical aluminum nitride (AlN)-based BAW filters. In this work, a modified lattice configuration is proposed to achieve a wideband filter response using AlN-based BAW resonators. The single stage of this novel topology comprises two auxiliary inductors paralleled in the balanced input and output of the conventional lattice topology. In multi-stage configuration, adjacent two auxiliary inductors can be combined into one; thus, the number of auxiliary inductors decreases exponentially, enabling the compact integration of filter chips. The circuit analysis is performed to reveal the working principle of this configuration. The systematic design methodology is developed ranging from the schematic design to the electromagnetic (EM) simulation. For proof-of-concept validation purposes, a prototype film bulk acoustic wave filter in this configuration is designed and fabricated. The measured 3-dB bandwidth is 400 MHz at the central frequency of 3.25 GHz (12.3% relative bandwidth), which demonstrates a huge superiority in contrast with the conventional ladder and lattice topologies.

## 1. Introduction

**Figure 1:**
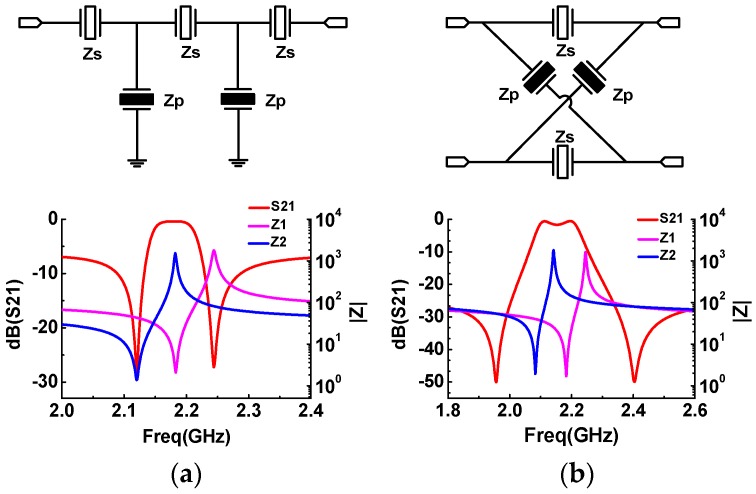
Main topologies of BAW filters, and their corresponding electrical responses: (**a**) ladder-type; and (**b**) lattice-type.

In recent years, the demand for advanced RF wireless communication systems with small size, high performance, and multifunction has facilitated the advent of various kinds of novel band pass filters [1,2,3]. Among them, AlN-based BAW filters gradually possess a dominant position, owing to their superior Q factors, high operating frequencies, enhanced power handling, and compatibility with the existing semiconductor processing [4,5,6]. Although the AlN-based BAW filters are sufficient to cover most of the current needs for the wireless communication systems, the demand for filters with large bandwidth, up to 10%, still remains a challenge for the conventional AlN-based BAW filters. Obviously, for a given filter topology, the maximal relative bandwidth of the filter is generally determined by the electromechanical coupling coefficient (*k_t_*^2^) of the resonators constituting this filter. In other words, the *k_t_*^2^ of BAW resonators and the selected filter circuit topology pose a limitation to the achievable bandwidth of the BAW filter. With the development of *c*-axis-oriented wurtzite AlN as a standard piezoelectric material for BAW resonators, the effective coupling coefficient of BAW resonators is on the order of 6%. On the other hand, two basic configurations are widely used in BAW bandpass filters [7]: the ladder-type topology and the lattice-type topology, as shown in Figure 1, which are composed of two kinds of resonators, *Z_s_* and *Z_p_*, placed in series and parallel arms, respectively. For the ladder topology, the parallel resonators are loaded to match their anti-resonant frequency with the resonant frequency of the series resonators so as to obtain a pass band characteristic, as shown in Figure 1a. For the lattice topology, a passband takes shape when one branch behaves inductively and the other behaves capacitively, while two transmission zeros are formed when all branches have equal impedance, as shown in Figure 1b. Inevitably, these two factors result in a moderate bandwidth of corresponding filters, typically less than 5%, which cannot meet the wide bandwidth (>10%) requirement.

In order to improve the bandwidth of the BAW filter, many methods and structures have been explored [8,9,10]. Two approaches are adopted currently. One is to add either serial or parallel inductors to each individual resonator of the filter [11], which enlarges the effective electromechanical coupling coefficient (*k_teff_*^2^) of each resonator, so as to broaden the bandwidth of the filter. However, the number of auxiliary inductors would increase manifold with the stage of the filter increasing, resulting in a substantially large volume of filter. Another method is to use an alternative piezoelectric material with higher inherent *k_t_*^2^ than AlN, such as PZT, lithium niobate (LiNbO_3_) [12,13,14,15], and co-doped AlN [16,17]. Nevertheless, the process of depositing new piezoelectric material is relatively immature and incompatible with the complementary metal oxide semiconductor (CMOS) process, which tends to degrade the performance and mass productivity of the filter. Given all that, these methods are not ideal for the realization of high-performance and cost-efficient wideband filters.

In this paper, a modified lattice topology with only two auxiliary inductors is reported to implement the wideband AlN-based BAW filters. Preliminary results on this topology were first demonstrated in [18]. It is the intention of this work to further investigate this configuration, including to present an in-depth theoretical analysis of its merits by comparing it with the other two conventional topology and to develop a systematical methodology for the design of this type of filters. This novel topology opens possibilities for more compact integration of filter chip by utilizing fewer inductors in contrast with the above-mentioned conventional method. In addition, the resonators employed in this topology are based on the reliant AlN piezoelectric thin film technique, which avoids the usage of new piezoelectric materials and maintains the outstanding performance in the insertion loss and out-of-band rejection of AlN-based BAW filters. In order to verify this configuration, a prototype filter was designed and manufactured. The measured 3-dB bandwidth was up to 12.3%, which was more than twice that of the conventional AlN-based BAW filter. 

## 2. Experimental Section

### 2.1. Working Principle of Novel Topology

**Figure 2:**
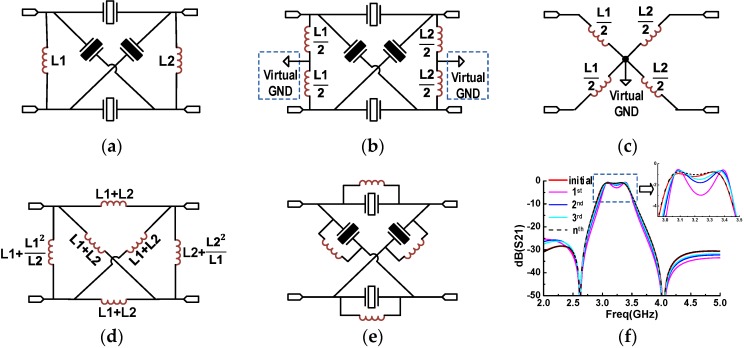
The theoretical analysis of modified lattice topology: (**a**) the proposed modified lattice configuration; (**b**) adding a virtual ground along the symmetrical axis of circuit; (**c**) a star circuit which is only composed of inductors; (**d**) an equivalent grid circuit; (**e**) the final equivalent circuit; and (**f**) the electrical response comparison between the equivalent circuit and initial circuit.

Figure 2a shows the circuit schematic of our proposed modified lattice configuration, which comprises two inductors paralleled in the input and the output port of the conventional lattice topology, respectively. In order to reveal the work principle of this topology, the equivalent circuits are derived step by step.

Since the circuit is driven differentially, a virtual ground can be established along its axis of symmetry. Accordingly, each shunt inductor is split equally into two series inductors, separated by the virtual ground (Figure 2b). To further simplify the analysis, the resonators are removed temporarily under the superposition principle of the circuit, leaving a star circuit that only consists of inductors, as illustrated in Figure 2c. The star circuit can be transformed to a grid circuit, which contains an inductor between every two ports (Figure 2d). It is found that the circuit construction in Figure 2d is quite similar to that in Figure 2a except the increasing values of shunt inductors connected to the input and the output. Thus, when the above equivalent transformation from Figure 2c to Figure 2d repeats several times, the values of these two inductors continue to increase, yet the values of the other four inductors connected between the input and output decrease gradually. After adequate cycles of the transformation, the impedance of the input and the output inductor is large enough so as to be omitted from the circuit. Then, the ultimate equivalent circuit is achieved, as shown in Figure 2e, which is generally called the enlarged lattice topology.

In Figure 2f, we compare the electrical responses of the initial circuit with the equivalent circuits undergoing different times of transformations. It is obvious that, with more cycles of transformation, the electrical response of the equivalent circuit is closer to that of the initial proposed circuit. Especially, in the case that the L1 is equal to the L2, the inductors in the final equivalent circuit would possess the same value of L1 and L2, which lays the foundation for the filter design discussed in the next part.

**Figure 3:**
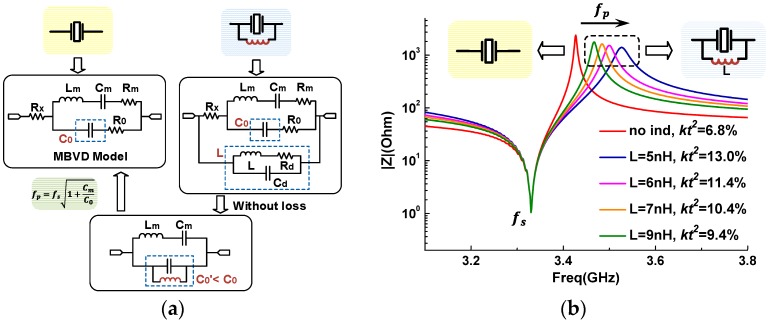
The impact of the parallel inductor on the frequency response of a resonator: (**a**) the equivalent MBVD model for the resonator with a parallel inductor; and (**b**) the simulated frequency responses with different values of inductor.

On the other hand, it is seen that this equivalent circuit is actually a conventional lattice topology, which contains a resonator and a parallel inductance in every arm. In order to analyze the impact of the inductor on the frequency response of each resonator, the Modified Butterworth-van Dyke (MBVD) circuit model [19] is introduced, which is a simple equivalent nonphysical model with a compact form. It consists of three reactive elements, *C_m_*, *L_m_*, and *C*_0_, determining the fundamental mode of the resonator, and three resistors, *R_m_*, *R*_0_, and *R_s_*, describing various bulk, perimeter, and parasitic losses, as shown in the upper left of Figure 3a. A sketch of the analysis procedure is outlined in Figure 3a. It starts from observing expressions of series and parallel resonance frequencies of the resonator:

(1) $$\omega_{s}=\frac{1}{\sqrt{L_{m}C_{m}}}$$

and:

(2) $$\omega_{p}=\omega_{s}\cdot \sqrt{1+\frac{C_{m}}{C_{0}}}$$

where the motional components, *C_m_* and *L_m_*, represent the electromechanical response of a piezoelectric material, the *C*_0_ is the electrical capacitance between the two electrodes. From the first equation, it is seen that the series-resonance frequency, *ω_s_*, is inversely proportional to the square root of the reactive elements of the motional arm, implying that it would keep constant with the addition of the parallel inductor. From the second equation, it indicates that the parallel-resonance frequency, *ω_p_*, varies inversely with the *C*_0_. When the *C*_0_ is paralleled with an extra inductor, *L*, as shown in the upper right of Figure 3a, the equivalent static capacitance at the frequencies around the *ω_p_* would be affected. Here a model of a single shunt capacitance across a lossy inductor is adopted, which has a self-resonant frequency at high frequency band. In fact, this distributed capacitance for the commercialized RF inductor, such as the LQP03T series from Murata (Kyoto, Japan) used in this work, is very tiny so that the resulting self-resonant frequency is much higher than the operating frequency of the resonator. Thus, this capacitance can be neglected at the following analysis. Furthermore, since the frequency variation induced by the extra inductor is scarcely influenced by the loss of the circuit, the resistors in the circuit could be eliminated, as shown in the bottom of Figure 3a. Then the corresponding admittance of the equivalent static capacitance takes the form as:

(3) $$Y\left(\omega \right)=j\omega C_{0}\prime =j\omega C_{0}\left(1-\frac{\omega_{0}^{2}}{\omega^{2}}\right)$$

where:

(4) $$\omega_{0}=\frac{1}{\sqrt{LC_{0}}}$$

It means that the auxiliary inductor would generate a new anti-resonance at *ω*_0_. Generally, the value of *L* needs to be set appropriately to make sure that the *ω*_0_ is much lower than the *ω_p_*. Then it would lead to a pseudo-passband of the lattice filter around *ω*_0_ to exacerbate the stop-band attenuation. However, the spurious passband can be suppressed to an acceptable level through the optimization of filters. When *ω* > *ω*_0_, the equivalent admittance is capacitive, and the value of this equivalent capacitance *C*_0_′ is smaller than *C*_0_*,* which finally results in the increase of the *ω_p_* of the resonator. 

**Figure 4:**
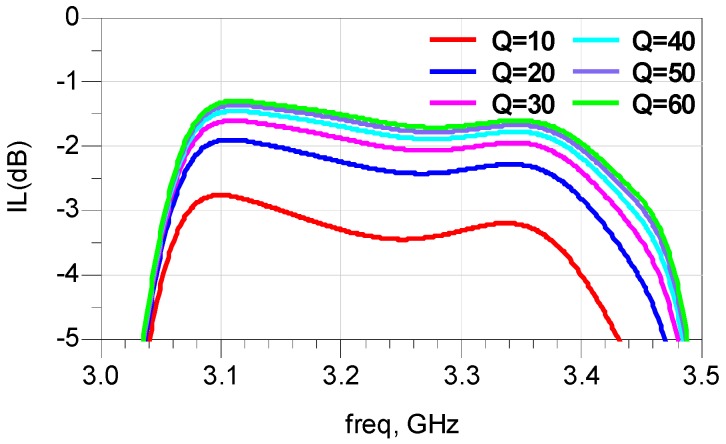
The simulation of the insertion loss of pass band with the different quality factor of auxiliary inductors.

The simulated relations between frequencies of a BAW resonator and values of *L* are intuitively illustrated in Figure 3b. Apparently, with a decrease in the value of *L* from 9 to 5 nH, the *k_teff_*^2^ of the BAW resonator increases notably from 9.4% to 13.0%. For a given topology, the maximal relative bandwidth attainable varies directly with the *k_teff_*^2^ of corresponding resonators. Hence, the maximal passband width achievable for this kind of filter will be remarkably enhanced. This indicates that the proposed modified lattice topology is competent to broaden the bandwidth of the filter. From the simulation result, it is also observed that the resistance at the parallel resonance (*R_p_*) decreases with the inductor added to the resonator. This can be explained by the ohmic dissipation induced by the inductor. The energy dissipation is quantified by the quality factor, *Q*, which is defined as *ωL*/*R_d_* for the inductor. The *Q* value of auxiliary inductor decreases with the increasing *R_d_*, and further deteriorates the insertion loss of the filter. The simulated insertion loss with different *Q* of inductors are shown in Figure 4. It shows clearly that the insertion loss becomes better with the rise of *Q* values. When *Q* values exceed 40, the promotion effect on the insertion loss is small.

**Figure 5:**
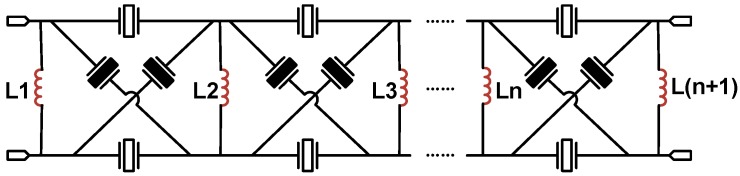
The multi-stage configuration of the modified lattice topology.

Although the bandwidth of the filter can be improved by applying either the modified lattice topology or its equivalent circuit, one should notice that the number of inductors in the modified lattice topology is half that in the equivalent circuit. The number of inductors would be dramatically reduced when this novel topology is employed in a muti-stage configuration, as shown in Figure 5. Clearly, the inductors paralleled in two facing ports have been combined into one equivalent inductor. It results that the number of inductors needed for the filter in the modified lattice topology is only one more than the number of stages. On the contrary, the number of inductors required for the filter in the enlarged lattice topology is four times that of stages. This demonstrates that the modified lattice topology is more promising to fulfill the trend of miniaturization and integration of RF filters.

### 2.2. Filter Design Method

Based on the above theoretical consideration, a two-stage lattice filter with three inductors is designed and optimized. To start with, according to the target bandwidth of the filter, and the relationship between the *k_teff_*^2^ of resonators and the maximal bandwidth of the filter in the lattice and ladder topology, we can roughly calculate the minimal required *k_teff_*^2^ of the single resonator. Then, we should judge whether the *k_teff_*^2^ is in the operable range of AlN-based resonators, and decide which kind of filter topologies should be adopted. If the calculated *k_teff_*^2^ is out of range, the modified lattice topology should be used. Next, according to the center frequency of the filter, we can choose a reasonable stack of the BAW resonator and obtain the corresponding native *k_t_*^2^. In order to reach the calculated *k_teff_*^2^, the parallel inductor is added like the branch in the equivalent lattice topology, as shown in Figure 2e. Finally, in light of the special case in the above part, the value of auxiliary inductors in the modified lattice configuration is equal to the parallel inductors in the equivalent lattice topology. Once the filter topology, the stack of resonators, and the initial values of inductors are determined, the Advanced Design System (ADS) software is used to optimize all parameters to achieve the target filter response. For the preliminary design, it is usually sufficient to use lumped elements to model the resonators, the bonding wires, and some laminate interactions in the ADS software.

Following the above steps, we employ the film bulk acoustic resonator (FBAR) [20] as the building elements of our filter without any loss of generality. FBAR is a particular type of BAW resonator which uses an air cavity instead of an acoustic mirror to decouple the resonator from the substrate. A typical FBAR can be viewed as a suspended structure composed of two metallic electrodes sandwiching a piezoelectric layer. The thickness of sputtered piezoelectric thin film and metal electrode layers together set the native resonance frequency. In order to design the filter operating at about 3 GHz, the corresponding thickness of the AlN piezoelectric layer is selected to be 7600 Å, and the thicknesses of the top and the bottom molybdenum (Mo) electrodes are 1500 Å. In the simulation, this individual resonator exhibits the series-resonance frequency in 3.33 GHz with the *k_teff_*^2^ of 6.8%.

Since the design environment is assumed to be free of the interference of electromagnetic parasitic and coupling effects [21], the schematic design above is not adequate to predict the actual performance of the filter. In practice, the filter is exposed to a time-varying electromagnetic field, which causes the parasitic inductance and capacitance existing between resonators and pads in the chip, as well as the electromagnetic coupling between bonding wires. These parasitic factors can severely degrade the performance of the filter, especially the distribution of transmission zeros in the stop band. Thus, an electromagnetic (EM) simulation is utilized to describe the laminate and evaluation board, bonding wires, and the non-acoustic parts of the filter die. It is expected to provide more accurate prediction of the device response, and to guide the design of the laminate or evaluation board.

**Figure 6:**
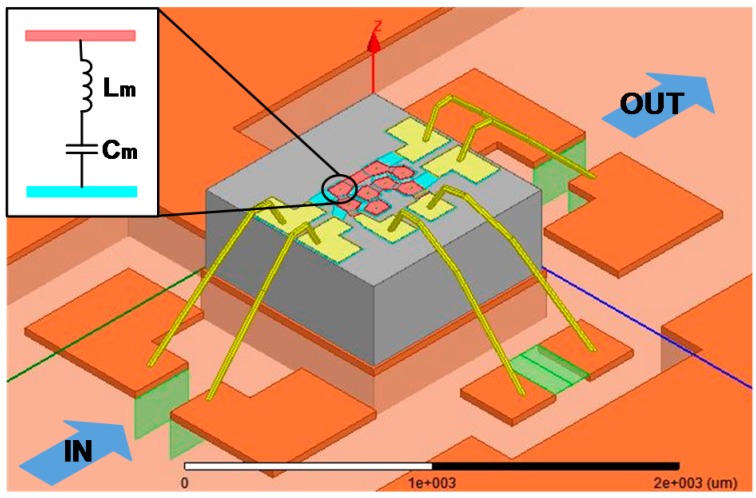
Electromagnetic model of the modified lattice filter based on FBARs.

Figure 6 illustrates a three-dimensional (3D) model of the filter chip built in High Frequency Structure Simulator (HFSS), including the bonding wires, and part of the evaluation board. In order to correctly describe the currents in the different resonators, each resonator contains a pair of ports between the top and bottom electrode, allowing for later connection to the acoustic branch in a Butterworth-van Dyke (BVD) model [22], as shown in the inset of Figure 6. On the other hand, instead of directly using the electromagnetic (EM) model of surface mounted inductors in the simulation, ports have been inserted in their place so that the tunable inductance can be added later in the circuit simulator. Then, the S-parameters from the full-wave EM-simulation is extracted and placed in the circuit simulator to gain a comprehensive model for the filter.

### 2.3. Comparison with Conventional Topologies

**Figure 7:**
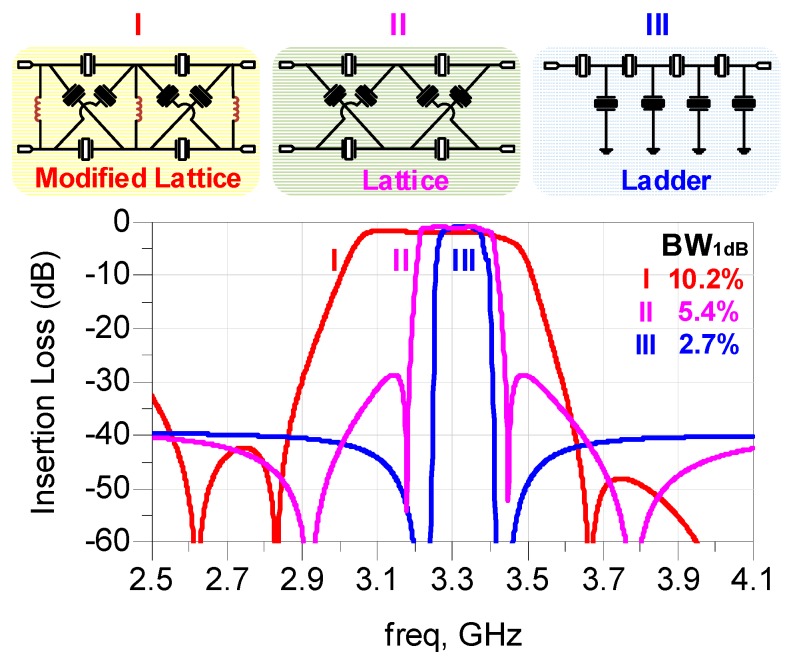
The comparison of simulated electrical responses among the modified lattice topology, the lattice topology, and the ladder topology.

In order to further evaluate the features of the modified lattice topology, we compare this topology with the other two conventional topologies, ladder topology and lattice topology. For a fair comparison in terms of achievable bandwidth among these topologies, the same numbers of resonators with the same *k_teff_*^2^ are employed to construct these three kinds of filters, which are labelled in the Figure 7 as I, II, and III, respectively. Although the relatively small inductance induced by the bonding wires are not depicted in the Figure 7, it has been taken into account in the actual simulation. In each filter topology, two important design parameters, the areas of resonators and the frequency spacing between the series and shunt resonators, are optimized to satisfy the rational targets set up in advance. The areas of resonators mainly affect the impedance matching characteristics in the pass band as well as the out-of-band rejection. The frequency spacing between the series and shunt resonators primarily influences the bandwidth and return loss of the pass band.

The final simulated schematic response of these three filters are shown in the bottom of Figure 7, with 1-dB relative bandwidth calculated to be 10.2%, 5.4%, and 2.7%, respectively. From the result, it is seen that each topology has its own pros and cons. The ladder structure achieves a high selectivity, but yields a relative low out-of-band rejection and a narrow bandwidth, whereas the lattice topology provides a larger out-of-band rejection and bandwidth at the expense of roll-off and return loss. The modified lattice topology inherits not only the merit of the lattice topology in the excellent out-of-band rejection but also the demerit in the poor roll-off, which would seriously deteriorate the shape factor. However, it presents an overwhelmingly large bandwidth over the other two kinds of filters.

On the one hand, although the modified lattice filter exhibits the larger bandwidth, its insertion loss is degraded in contrast with the other two types of filters. We attribute it to the decrease of the resistance at the parallel resonance (*R_p_*) of shunt resonators affected by the auxiliary inductors. This fact is directly reflected by the cluster of electrical response curves in Figure 3b that the *R_p_* is indeed decreasing with the *k_teff_*^2^ increasing. Accordingly, there is a tradeoff between the bandwidth and the insertion loss in the modified lattice topology. However, this tradeoff could be circumvented to a certain extent by improving the performance of the resonators.

## 3. Results and Discussion

**Figure 8:**
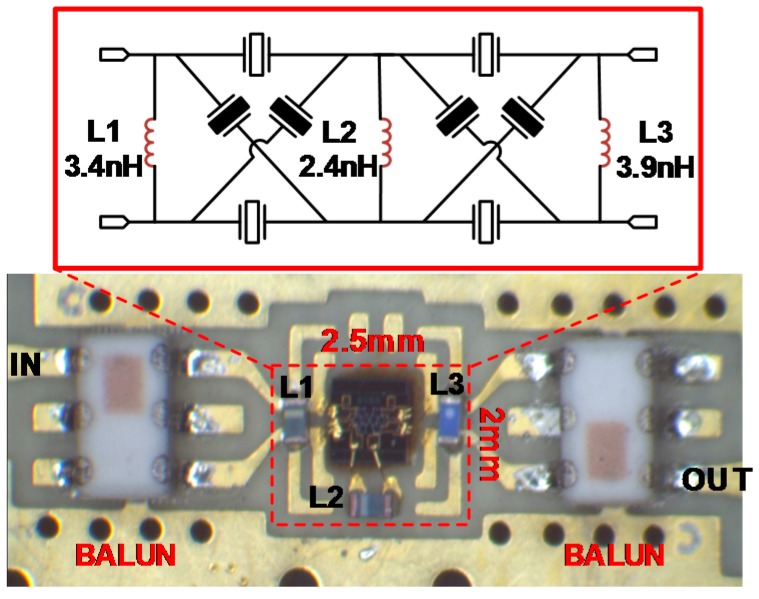
Design topology and board assembly of the filter with the proposed architecture.

According to the above method, a prototype filter is designed in this modified lattice topology, as shown in the top row of Figure 8. The values of three auxiliary inductors are 3.4, 2.4, and 3.9 nH, respectively. The filters are mass fabricated by a CMOS-compatible Micro-electromechanical Systems (MEMS) process in a high-resistivity silicon wafer. The single die size is 1 mm × 1 mm. The fabrication process starts by etching an air cavity directly on the silicon wafer by deep reactive ion etching (DRIE). Then it is filled with phosphosilicate glass (PSG), as a sacrificial layer, by chemical vapor deposition (CVD). Next the deposited wafer was reduced to expose the silicon surface again by chemical mechanical polishing (CMP). Afterward Mo is deposited and patterned as the bottom electrode. Then follows depositing AlN and Mo by physical vapor deposition (PVD), successively, to form the piezoelectric layer and top electrodes, respectively. The AlN film shows a strong c-axis orientation with the full width at half maximum (FWHM) measured to be 1.657. The top electrode is then patterned by plasma etching, while AlN is etched by KOH wet etching. Finally, gold (Au) is evaporated by PVD and patterned by lift-off, serving as the electrical connection and pads, which is followed by the release of the sacrificial layer in diluted hydrofluoric acid solution (HF/H_2_O, 1:10).

As the wafer is fabricated, the desirable filter dies are identified through the on-wafer test and picked up for further assembly. For quick demonstration, it is directly mounted on a prepared evaluation board along with three 0201 sized surface mounted inductors by non-conductive epoxy. The bonding pads on the filter die are electrically connected to the corresponding pads in the EVB via bonding wires. Two ceramic baluns, NCS2-83+, from Mini-Circuit, are placed in the input and output port of the filter, respectively, which serve to convert each balanced port to a single-ended port in order to measure this network by a two-port vector network analyzer (VNA). A typical implemented filter is displayed in the bottom row of Figure 8. The area of the complete filter, including the external inductors, is 2.5 mm × 2 mm. Note that the filter volume could be further reduced by shrinking the layout area of filter die and properly designing the laminate with peripheral inductors buried in it.

**Figure 9:**
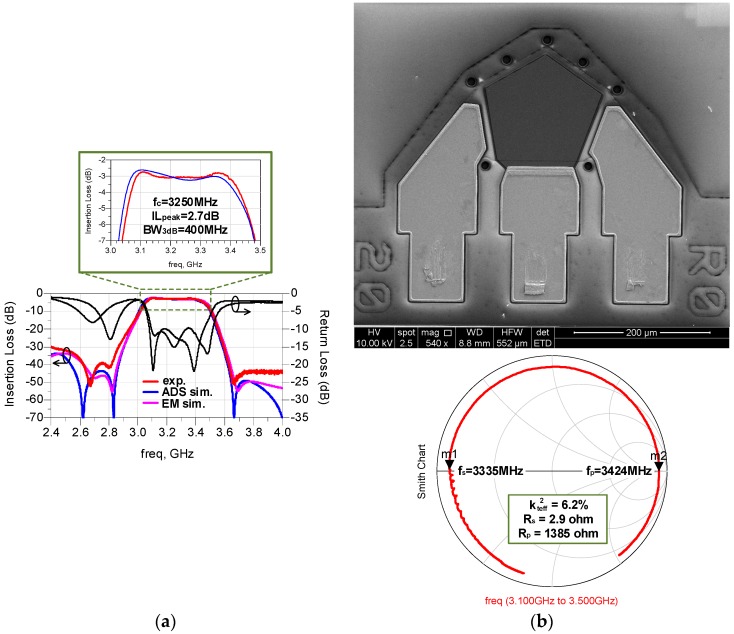
(**a**) The frequency response of the fabricated filter vs. simulation results; (**b**) the scanning electron microscope (SEM) image of 50-Ω FBAR and its corresponding Smith chart.

The electrical responses of the filter from both experiment and simulation are plotted in Figure 9a. The red and black curves are based on the measurement data, the blue one is the schematic simulation result, and the magenta one is the EM simulation result. It is worth mentioning that a through, reflect, line (TRL) calibration is performed before measurement for the sake of accuracy. Since not all the acoustic loss has been included in the method of the EM simulation, the passband characteristic of the EM simulation is not credible. Thus, in the zoomed-in view of the passband, we merely compare the experiment data and the schematic-simulated response, which demonstrates good agreement with each other. The peak value of the insertion loss is less than 3 dB, and the out-of-band rejection is more than 30 dB. The 3-dB bandwidth is 400 MHz at a center frequency of 3.25 GHz with the return loss larger than 10 dB, indicating a relative bandwidth larger than 12%. 

Although the EM simulated stopband performance is very close to the measured response, there still exist some discrepancies between the measurements and simulations in the stopband. It may be caused by the baluns, parasitic electromagnetic interference, and coupling effects excluded in the EM model. This interference can be diminished by fine-tuning the bonding wires and further optimizing the filter design and the method of assembly. In addition, the introducing of baluns will have a negative impact on the actual performance of filter, such as degrading the insertion loss. However, this problem can be solved completely by using a four-port measurement system, which could measure the full-balanced device directly without the need of baluns to realize the single-ended to differential conversion. 

The scanning electron microscope (SEM) image of a 50-Ω FBAR and its corresponding Smith chart is shown in Figure 9b. The *k_teff_*^2^ of the resonator is measured to be 6.3%. Thus, the calculated values of maximal relative bandwidth attainable for filters in the lattice and ladder topology are 5% and 2.5%, respectively. They are significantly smaller than the relative bandwidth of filters in our proposed configuration. Thus, the modified lattice topology successfully demonstrates an advanced solution for the wide-bandwidth BAW filters. 

## 4. Conclusions

This work presents a modified lattice topology to accomplish the wideband AlN-based BAW filter with fewer numbers of auxiliary inductors. The equivalent circuit is derived to reveal the working principle of this novel topology. Compared with the equivalent circuit, the chip size is effectively reduced with this topology. In order to bear out the feasibility of the proposed topology, a prototype FBAR filter employing this configuration is designed and fabricated. The measurement results of the filter present a 12% relative bandwidth at the 3.25 GHz central frequency, which marks a significant improvement to the current BAW filter products [23,24].
